# Detection of a Double-Stranded MGMT Gene Using Electrochemically Reduced Graphene Oxide (ErGO) Electrodes Decorated with AuNPs and Peptide Nucleic Acids (PNA)

**DOI:** 10.3390/bios12020098

**Published:** 2022-02-05

**Authors:** Mina Safarzadeh, Genhua Pan

**Affiliations:** Wolfson Nanomaterials and Devices Laboratory, School of Engineering, Computing and Mathematics, Faculty of Science and Engineering, University of Plymouth, Plymouth PL4 8AA, UK; g.pan@plymouth.ac.uk

**Keywords:** electrochemical reduction of graphene oxide, rGO, PNA, detection of double-stranded DNA, MGMT

## Abstract

The ability to detect double-stranded DNA (dsDNA) as a biomarker without denaturing it to single-stranded DNA (ss-DNA) continues to be a major challenge. In this work, we report a sandwich biosensor for the detection of the ds-methylated MGMT gene, a potential biomarker for brain tumors and breast cancer. The purpose of this biosensor is to achieve simultaneous recognition of the gene sequence, as well as the presence of methylation. The biosensor is based on reduced graphene oxide (rGO) electrodes decorated with gold nanoparticles (AuNPs) and uses Peptide Nucleic Acid (PNA) that binds to the ds-MGMT gene. The reduction of GO was performed in two ways: electrochemically (ErGO) and thermally (TrGO). XPS and Raman spectroscopy, as well as voltammetry techniques, showed that the ErGO was more efficiently reduced, had a higher C/O ratio, showed a smaller crystallite size of the sp^2^ lattice, and was more stable during measurement. It was also revealed that the electro-deposition of the AuNPs was more successful on the ErGO surface due to the higher At% of Au on the ErGO electrode. Therefore, the ErGO/AuNPs electrode was used to develop biosensors to detect the ds-MGMT gene. PNA, which acts as a bio-recognition element, was used to form a self-assembled monolayer (SAM) on the ErGO/AuNPs surface via the amine-AuNPs interaction, recognizing the ds-MGMT gene sequence by its invasion of the double-stranded DNA and the formation of a triple helix. The methylation was then detected using biotinylated-anti-5mC, which was then measured using the amperometric technique. The selectivity study showed that the proposed biosensor was able to distinguish between blank, non-methylated, non-complementary, and target dsDNA spiked in mouse plasma. The LOD was calculated to be 0.86 pM with a wide linear range of 1 pM to 50 µM. To the best of our knowledge, this is the first report on using PNA to detect ds-methylated DNA. This sandwich design can be modified to detect other methylated genes, making it a promising platform to detect ds-methylated biomarkers.

## 1. Introduction

DNA methylation is the most exhaustively characterized epigenetic alteration of DNA in which methyl groups (CH_3_) are covalently bound to DNA. This alteration predominantly happens on cytosines preceding guanines (CpG sites) [1]. The aberrant methylation of the CpG sites has the potential of being a diagnostic, prognostic, and predictive biomarker for various diseases [2,3] including lung cancer [4,5], brain tumors [6], breast cancer [7,8], and prostate cancer [9,10]. The conventional techniques for the detection of DNA methylation are based on bisulfite treatment, methylation-specific PCR (MSP), mass spectrometry (MS), and liquid chromatography (LC), all of which are highly sensitive. However, these techniques have some limitations, such as requiring expensive equipment and significant sample sizes, as well as specific expertise [11,12,13]. On the other hand, biosensors have the potential to overcome these limitations due to advantages such as operational simplicity, portability, low cost, and rapid detection. They can either be used on their own or can be combined with conventional techniques to advance the assay specificity and sensitivity. DNA sequencing techniques are another approach for overcoming the limitations of the conventional techniques [11]. 

Wang et al. [14] developed an electrochemical assay for the detection of circulating methylated DNA based on a sequential discrimination-amplification strategy (SEDA). In this assay, the methylated DNA first underwent a bisulfite modification and then was identified and amplified using asymmetric MSP (AMSP). Finally, it was hybridized with tetrahedral DNA probes that were decorated on a gold electrode. Avidin–HRP was used as the label for amperometric detection. The dynamic range for this assay was reported to be 3–150 pg and the LOD was one methylated DNA molecule in the presence of a 1000-fold excess of unmethylated alleles. Povedano et al. [15] reported an electrochemical platform to detect the four most frequent methylations in DNA and RNA (5mC, 5-hmC, 6mA, and m6A). In this work, the target biomarkers were first captured on protein G-modified MBs (ProtG-MBs) using the corresponding capture antibody for each methylation (anti-5-mC, anti-5-hmC, or anti-m6A/6mA). Subsequently, the amperometric detections were performed using screen-printed electrodes with four carbon working electrodes (SP_4_CEs) and streptavidin-HRP as the label. The linear ranges were reported to be 3.9 × 10^−4^–1.9 μM, 2.3 × 10^−4^–1.8 × 10^−1^ μM, 5.4 × 10^−4^–1.1 × 10^−1^ μM, 1.7 × 10^−5^–3.5 × 10^−1^ μM with LOD of 3 × 10^−5^ μM, 3 × 10^−5^ μM, 1 × 10^−4^ μM, 9 × 10^−7^ μM for 5-mC, 5-hmC, 6mA, and m6A, respectively. Chen et al. [16] developed an electrochemical biosensor for DNA methylation detection using tetrahedron DNA probes which were anchored to a AuNPs-coated gold electrode with avidin-HRP as the label. This biosensor showed a dynamic range of 1 aM to 1 pM, with the LOD of 0.93 aM.

Peptide Nucleic Acid (PNA) is an artificially synthesized nucleic acid analogue with N-(2-aminoethyl)-glycine motif backbones which are linked together via peptide bonds [17]. PNAs have been used as a bio-recognition element in biosensors as a replacement for DNA-based probes, antibodies, or enzymes to overcome limitations such as denaturation during the assay and steric hindrance caused by large molecules [18]. PNA displays many advantages, including specificity, versatility, and neutral charge, as well as high chemical, biological, and thermal stability. PNA/DNA complexes are shown to be more stable than DNA/DNA systems, with PNA probes being more efficient in hybridization because of its complementary target sequence that leads to an enhanced assay sensitivity [17,19]. Besides superior specificity towards ssDNA and RNA, PNA has shown the ability to specifically target the sequence of dsDNA by strand invasion, forming a triplex structure [20].

Hamidi-Asl et al. [21] reported a PNA-based biosensor for the electrochemical detection of the point mutation of the p53 gene. In this work, first, thiolated PNA probes formed a SAM on a gold electrode surface and then the electrode was incubated in the ds-target gene to form triplex structures. Methylene blue (MB) was used as the label to enhance the electrochemical signal. The linear range was reported to be 10 pM to 5 × 10^7^ pM with an LOD of 4.15 pM. Ahmadi and Ahour [22] developed a biosensor based on a graphene oxide modified pencil graphite electrode and PNA to electrochemically detect dsDNA in plasmid samples. GO was first casted on to the pencil graphite electrode and then the PNA probes were immobilized on the modified electrode. Upon incubation of the biosensor in the target ds-DNA, PNA probes detached from the electrode surface, resulting in a guanine oxidation signal, decreasing linearly with the target concentration. Under optimized conditions, the linear range was from 30 pM to 10 nM and the LOD was reported to be 1.3 pM. Ahour et al. [23] reported an electrochemical biosensor for the detection of the double-stranded plasmid (ds-Pl) using PNA probes and a gold electrode. The PNA oligomer probes were first immobilized on the surface before capturing the ds-PI target, forming a PNA/ds-PI structure using MB as the label. The dynamic range was from 10 to 300 pg/μL with an LOD of 9.5 pg/μL.

In this work, the surface of the working electrode was first drop-coated with GO, followed by a reduction of GO using two different methods (electrochemically and thermally). XPS and Raman spectra showed that ErGO was more efficiently reduced and was more stable using voltammetric measurements than TrGO. After that, AuNPs were electro-deposited on both rGO surfaces. XPS spectra showed that the At% of Au on the ErGO was the highest. SEM and EDS studies confirmed the presence of AuNPs on the ErGO surface. Therefore, the ErGO/AuNPs electrode was used to develop the biosensor assay with the aim of detecting ds-MGMT. Therefore, PNA was used to invade the duplex of the target gene to create a triple helix, after which the methylated sites of the captured ds-MGMT gene were detected using biotinylated-anti-5mC, which were then measured amperometrically using Streptavidin-HRP as the label. The LOD was calculated to be 0.86 pM with a wide linear range, and the developed biosensor showed a high sensitivity in mouse plasma. This biosensor is the first report for the detection of the double-stranded methylated gene, is bisulfite- and PCR-free, and can be tailor-made to detect other methylated genes, which can be beneficial in point-of-care (POC) programs as an inexpensive platform to detect methylated DNA biomarkers.

## 2. Experimental

### 2.1. Materials and Methods

All of the reagents used in this work were of analytical grade. Potassium ferricyanide (K_3_[Fe(CN)_6_]), potassium chloride (KCl), hydroquinone (HQ), hydrogen peroxide solution 30% (H_2_O_2_), Nuclease-Free Water, gold(III) chloride trihydrate (HAuCl_4_), sulphuric acid (H_2_SO_4_), and 6-Mercapto-1-hexanol (MCH) were purchased from Sigma-Aldrich (Gillingham, UK). Single-layer graphene oxide solution (GO) was ordered from Graphene Supermarket (Ronkonkoma, NY, USA). PNA was obtained from Cambridge Research Biochemicals (Billingham, UK) and its sequence was N-AEEA-AEEA-CACCAAGTCGCAAACGGTGC-C. All other nucleic acids were purchased from Integrated DNA Technologies (Coralville, IA, USA). The ds-MGMT target gene sequences were as follows: GTCCC(M)GAC(M)GCCC(M)GCAGGTCCTC(M)GCGGTGCGCACCGTTTGCGACTTGGTG and CACCAAGTCGCAAACGGTGCGCACCGCGAGGACCTGCGGGCGTCGGGAC, where C(M) was methylcytosine. The non-methylated target sequences were similar to ds-MGMT, with cytosine replacing methylcytosine. The non-complementary target was a methylated three-base mismatch of the target ds-MGMT. Biotinylated anti-5mC and Streptavidin-HRP were ordered from Abcam (Cambridge, UK). Mouse plasma was purchased from Sigma-Aldrich (UK). PBS tablets pH 7.4 were obtained from Fisher Scientific (Loughborough, UK) and the PBS buffer solution was prepared in Milli-Q water.

### 2.2. Apparatus and Measurements

The electrochemical measurements were performed using a µStat ECL BiPotentiostat/Galvanostat and commercially available screen-printed electrodes (SPE). The working electrodes of the SPEs were made of rGO, the counter electrodes were carbon, and the reference electrodes were silver. The electrodes and BiPotentiostat/Galvanostat were purchased from DropSens (Asturias, Spain).

The CV voltammograms were obtained by cycling the potential between 0.55 and −0.2 V using a scan rate of 50 mV/s in 100 µL of 10 mM PBS pH 7.4 solution containing 10 mM K_3_[Fe(CN)_6_] and 1 M KCl as electrolyte agents. Amperometric measurements were performed in PBS containing 1 mM HQ under agitation at −0.2 V. Once the background current was stabilized, 0.1 M H_2_O_2_ solution was added and the current was recorded until the steady-state current was reached. The entire measurement was done in ~150 s. 

Raman spectra were obtained using an XploRA HORIBA system equipped with an Olympus BX41 microscope and a 532 nm green laser source. The spectra were acquired with a power of 100 mW, a scan range of 1100 to 3000 cm^−1^, and an exposure time of 5–60 s.

XPS analyses were carried out using a Thermo Scientific Nexsa X-Ray Photoelectron Spectrometer System with a monochromatic Al KXα-ray source (1486.68 eV) to obtain the spectra. The pass energy for wide scans was 200 eV with an energy step size of 1 eV and 10 scans. The pass energy for high resolution scans was 40 eV with an energy step size of 0.1 eV and 20 scans. The C/O ratios were calculated using the total At% of C1 peak divided by the total At% of O1s peak obtained from the XPS survey scan for each sample. All of the measurements mentioned above were carried out at room temperature.

### 2.3. Electrode Modification

**Figure A1:**
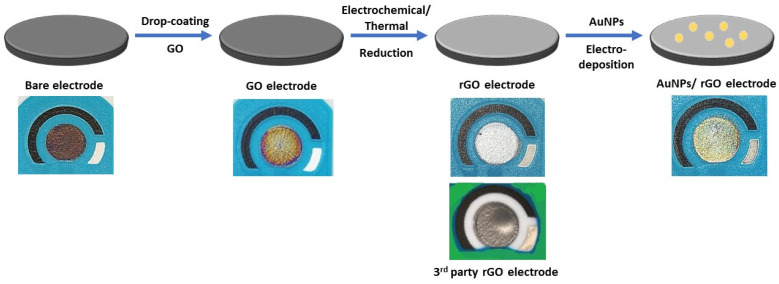
A schematic of the surface modification procedure of a screen-printed electrode (Section 2.3) and the changes to the appearance of the working electrode.

With the aim of increasing the reproducibility of the SPEs, the electrodes were modified using rGO and AuNPs. The working electrodes were first drop-coated with 0.15 mg/mL of GO aqueous solution and were left to dry at room temperature for 3 h. After that, the GO layers were reduced in two different ways in order to compare the impact of the reduction techniques on the quality of rGO and AuNPs. The electrodes were either reduced electrochemically (ErGO) or thermally (TrGO). The electrochemical reduction of GO was performed using 10 successive CV scans in 10 mM PBS pH 7.4 solution containing 10 mM K_3_[Fe(CN)_6_] and 1 M KCl over a potential range of 0.5 and −1.5 V and a scan rate of 100 mV/S. Thermal reduction was performed at 250 °C for 1 h. After reducing GO, the AuNPs were electro-deposited on both ErGO and TrGO electrodes from 0.5 mM H_2_SO_4_ solution containing 1 mM HAuCl_4_, using 5 successive CV cycles over a potential range of 1 and −1 V and a scan rate of 50 mV/s (Figure A1). The electrochemical reduction of GO and electro-deposition of the AuNPs were both performed at room temperature.

### 2.4. Assay Development

**Figure 1:**
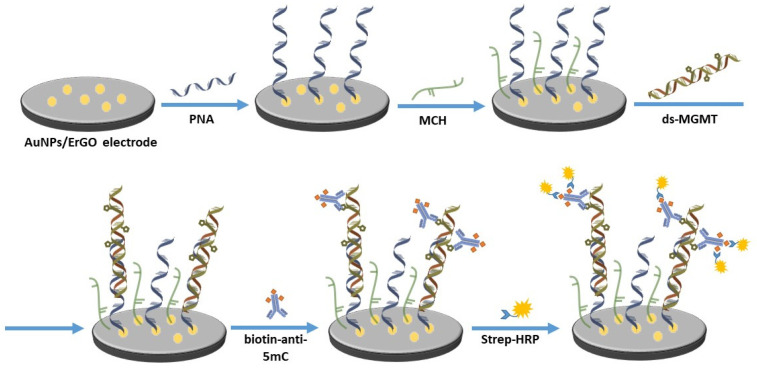
A schematic of the developed biosensor. The preparation of the AuNPs/ErGO electrode is depicted in Figure A1.

Modified electrodes were incubated in 10 µM PNA overnight at 4 °C to functionalize a self-assembled monolayer (SAM) on the surface of the working electrodes. On the next day, the electrodes were incubated in 1 mM aqueous solution of MCH for 5 min to minimize the nonspecific binding and then they were incubated in PBS for 1 h to stabilize the SAM. The prepared biosensors were then incubated in various concentrations of dsDNA for 1.5 h at 37 °C, followed by incubation in biotinylated methyl binding antibody for 2 h at room temperature. In order to use the amperometric technique, the biosensors were incubated in diluted Streptavidin-HRP for 30 min prior to the measurements. Various layers of the biosensor are depicted in Figure 1. After each incubation step, the electrodes were rinsed with ultrapure water. All of the incubation steps were carried out in a high humidity chamber. 

## 3. Results and Discussion

### 3.1. GO Reduction

GO-coated electrodes were reduced either electrochemically or thermally in order to achieve a higher reproducibility and quality. The reduction process for both ErGO and TrGO were described in Section 2.3. The reduction degree and the quality of the rGO-modified electrodes were compared using XPS, Raman, and cyclic voltammetry techniques.

**Figure A2:**
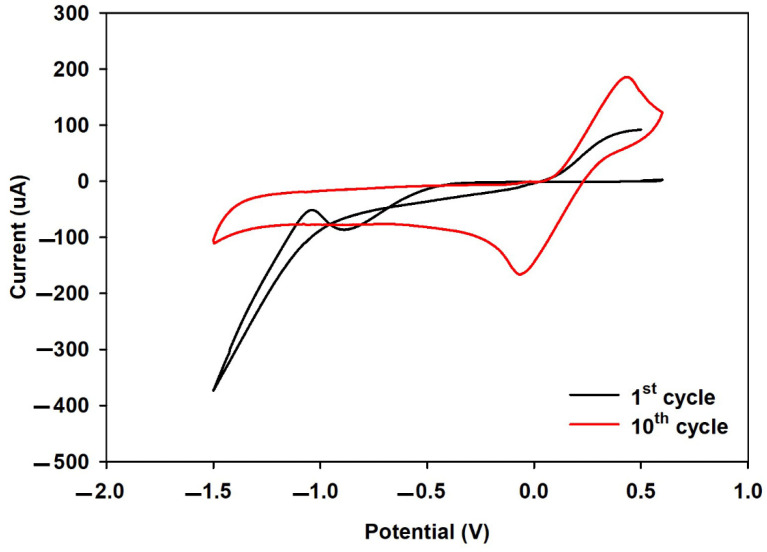
The voltammograms of the CV cycles used to electrochemically reduce GO.

The electrochemical reduction of GO was obtained using CV scans for 10 successive cycles. The voltammograms of reducing a GO modified electrode are shown in Figure A2. As can be seen in this figure, a large cathodic peak is located between −1.0 and −1.5 V, disappearing after several cycles. This peak can be attributed to the electrochemical reduction of the functional groups, mainly oxygenated groups which are present at the GO basal plane [24,25,26,27]. Therefore, after a few CV cycles, GO was reduced, rGO was obtained, and subsequently, the electric properties improved [28]. The reduction of GO can also be seen from the color change in the working electrode, which changes from black to silver after reduction, consistent with a third-party rGO electrode (Figure A1).

#### 3.1.1. XPS Measurements

**Figure 2:**
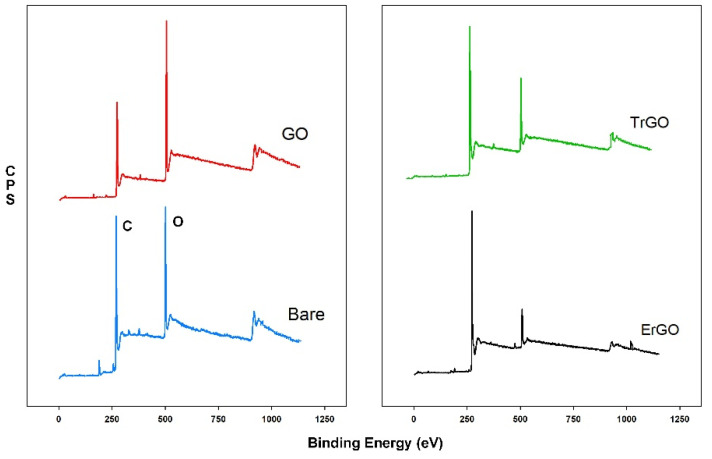
XPS wide scan spectra of a bare electrode and GO-, ErGO-, and TrGO-modified electrodes. The C/O ratios of these electrodes were 3.97, 2.49, 10.52, and 5.7, respectively.

**Figure A3:**
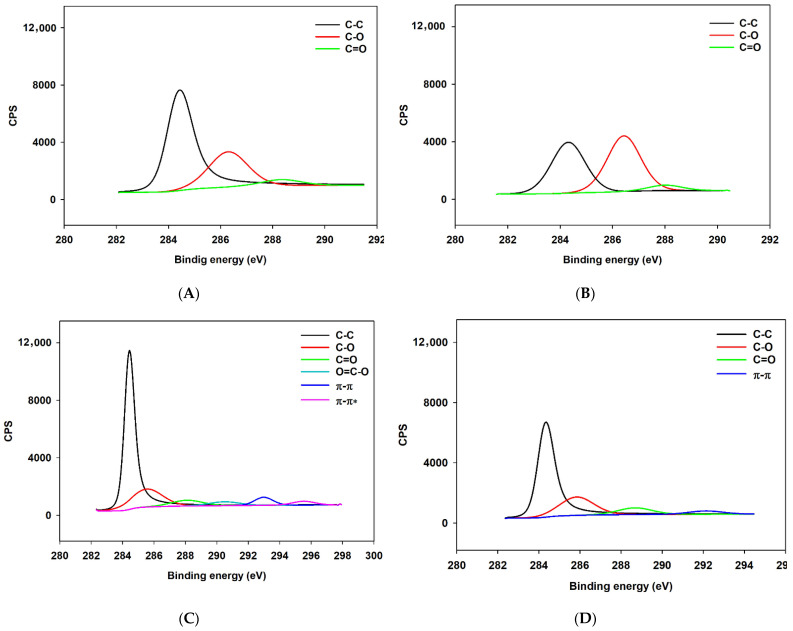
The C1s high resolution spectra of a bare electrode (**A**) and GO- (**B**), ErGO- (**C**), and TrGO- (**D**) modified electrodes.

XPS measurements were carried out in order to characterize and evaluate the chemical composition of a bare electrode and the GO-, ErGO-, and TrGO-modified electrodes. Wide scans (survey scans) as well as C1s high-resolution scans of all of the samples are shown in Figure 2 and Figure A3 respectively. Table A1 shows a detailed information for all of the peaks observed by the wide scan (Figure 2), their position, FWHM, and At%.

As can be seen in Figure 2, the survey scan spectra of all of the samples show the presence of carbon and oxygen and a trace of contaminants (Na, Cl, S, N), all less than 3% At%. The C/O ratios for the bare, GO, ErGO, and TrGO electrodes were calculated to be 3.97, 2.49, 10.52, and 5.7, respectively. Schniepp et al. [29] reported that in temperatures below 500 °C, the C/O ratio only reached 7; however, if the temperature was increased to 750 °C, the C/O ratio would rise to more than 13. Ren et al. [30] reported the C/O ratio ranging from 3.1 to 15.1, where the latter was obtained by reducing GO in 95 °C for 3 h using hydrazine hydrate as a reducing agent. Yang et al. [31] reported a C/O ratio in the range of 3.09 to 5.38 for the rGO samples that were reduced by adding NaBH_4_ and CaCl_2_ as catalysts and stirring for 12 h at room temperature. Chua et al. [32] increased the C/O ratio from 3.0 to 16.0 by using thiourea dioxide (CH_4_N_2_O_2_S) for 2 to 5 h at 90 °C. Although the C/O ratio of the ErGO reported in this study was not as high as the ones reported above, the electrochemical reduction of GO does not require a high temperature or any dangerous reductants. In addition, the reduction process for each electrode took less than 5 min.

Figure A3 shows the high-resolution C1s spectra of bare, GO, ErGO, and TrGO electrodes, where all of the spectra showed asymmetrical shapes. The C1s spectra of the bare electrode (Figure A3A) can be deconvoluted into three component peaks: a C-C peak located at 284.37 eV, a C-O peak at 286.29 eV, and a C=O peak located at 288.34 eV. The C1s spectra of the electrode covered with GO (Figure A3B) can be deconvoluted into a C-C peak located at 286.43 eV, a C-O peak at 284.31, and a C=O peak at 288.01 eV. The GO sample exhibited the highest amount of oxygen among the samples in both the wide scan and the C1s high-resolution scan. The C1s spectra for ErGO and TrGO exhibited a few tailing peaks. As can be seen in Figure A3C for the ErGO sample, a C-C peak is located at 284.40 eV, a C-O peak is at 285.55 eV, a C=O peak is at 288.10 eV, and a O=C-O peak is located at 290.49 eV. Two π-π peaks were also observed at 292.99 eV and 295.53 eV, respectively. The C1s spectra for the TrGO is shown in Figure A3D. The spectra can be deconvoluted into the following peaks: a C-C peak located at 284.29 eV, a C-O peak at 285.84 eV, and a C=O peak at 288.68 eV. A π-π peak was also observed at 292.14 eV [33,34,35].

#### 3.1.2. Raman Spectroscopy

**Figure 3:**
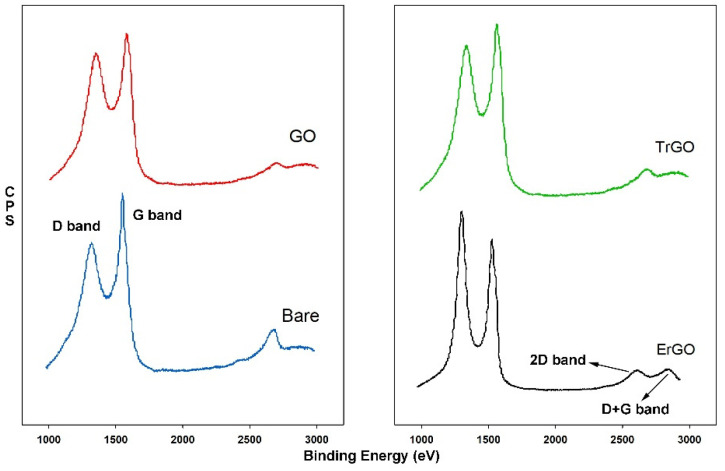
Raman spectra obtained from a bare electrode and electrodes modified with GO, ErGO, and TrGO. The I_D_/I_G_ ratios were 0.77, 0.88, 1.15, and 0.89, respectively.

Raman spectroscopy was carried out for a bare electrode and GO, ErGO, and TrGO coated electrodes, and the spectrographs are shown in Figure 3. The peaks at around 1570 cm^−1^ are G bands which are attributed to in-plane vibrations of the sp^2^-bonded graphitic carbon atoms. The peaks at 1350 cm^−1^ are D bands and represent the out-of-plane vibration of the disordered structures [36]. I_D_/I_G_ ratio, or the intensity ratio, is normally used to evaluate disorder level, or the ratio of structural defects in the GO or rGO layers. I_D_/I_G_ was calculated to be 0.77, 0.88, and 0.89 for bare, GO, and TrGO electrodes, respectively, but 1.15 for ErGO. The higher number in the intensity ratio of ErGO indicates that the reduction process may change the GO structure, resulting in an increase of defects in the structure and a decrease in the average size of the sp^2^ due to the removal of the oxygenated functional groups [37,38,39]. A 2D band and a D + G band, which become significant in rGO, were also observed at 2680 cm^−1^ and 2910 cm^−1^, respectively, demonstrating the restoration of the graphite structures [39]. The experimental values of the peak locations, I_D_/I_G_ ratios, and the average crystallite sizes of the sp^2^ lattice (L_a_) are listed in Table A2. The L_a_ values were calculated using the equation L_a_ (nm) = (2.4 × 10^−10^)λ_laser_^4^(I_D_/I_G_)^−1^ for all samples, where λ_laser_ is the laser wavelength and I_D_ and I_G_ are the intensities of the D and G Raman bands, respectively [25].

#### 3.1.3. Cyclic Voltammetry

**Figure A4:**
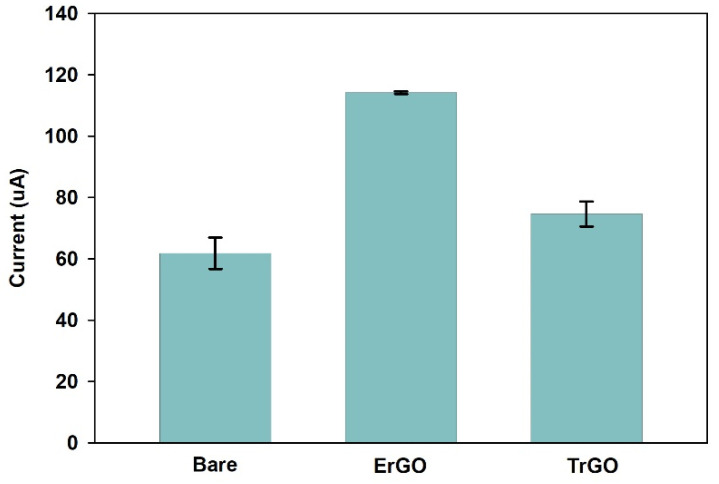
The anodic peak currents (I_pa_) of 10 successive cycles of CV voltammograms of a bare electrode and electrodes modified with ErGO and TrGO.

Cyclic voltammetry was used to compare the stability of the electrodes during electrochemical measurements. 10 successive CV scans were performed for a bare electrode, an ErGO-, and a TrGO-modified electrode and the average of the anodic peak currents (i_pa_) of the voltammograms was plotted in Figure A4. As can be seen in this figure, the ErGO reached a higher current compared to both the TrGO and the bare electrodes. For 10 successive measurements, ErGO showed a higher stability and lower fluctuation in the peak currents.

### 3.2. AuNPs

**Figure A5:**
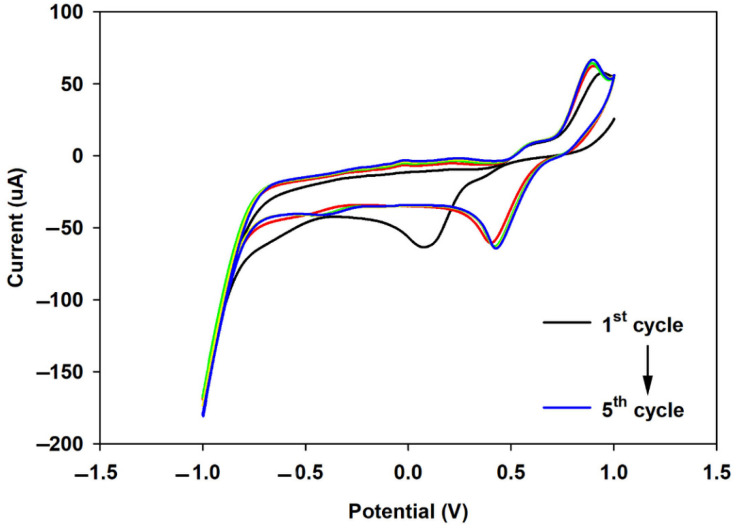
The voltammograms of five successive CV cycles used to deposit AuNPs.

AuNPs were electrochemically deposited on the surface of ErGO- and TrGO-modified electrodes and were characterized using XPS and EDS spectroscopy. SEM was also used to confirm the presence of AuNPs on the surface. The electro-deposition method was described in the experimental section (see Section 2.3). Figure A5 shows the voltammograms of the deposition process in five successive cycles with the potential range of −1 to 1 V. As can be seen in this figure, a cathodic peak is located at 0.1 V during the first scan, which can be attributed to the reduction of Au^3+^ ions to Au, as well as the seeding of the AuNPs. This peak has shifted to 0.5 V in the next cycles, indicating the easier electro-deposition of gold and the growth of the AuNPs. The Anodic peak at 0.8 V can be ascribed to the surface oxidation of the AuNPs [40,41,42].

#### 3.2.1. XPS Measurements

**Figure 4:**
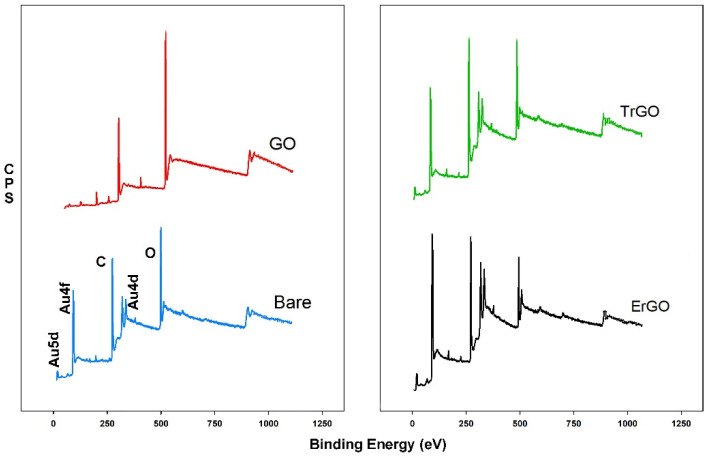
XPS spectra of the bare electrode and electrodes modified with GO, ErGO, and TrGO after the deposition of the AuNPs. The ErGO showed the highest At% for Au.

Figure 4 shows a wide scan XPS spectra of a bare electrode and the GO-, ErGO-, and TrGO-modified electrodes after the immobilization of the AuNPs. These electrodes are named bare/AuNPs, GO/AuNPs, ErGO/AuNPs, and TrGO/AuNPs, respectively. The bare/AuNPs wide scan spectrum showed two Au4f peaks centered at 83.83 eV and 87.81 eV and two Au4d peaks at 334.55 eV and 355.27 eV. A very small peak at 4.9 eV was also observed, attributed to Au5d. In the GO/AuNPs spectrum, no peaks related to the AuNPs were observed. However, the ErGO/AuNPs spectrum showed two Au4f peaks at 94.99 eV and 98.87 eV, respectively, as well as two Au4d peaks located at 351.1 eV and 369.24 eV. An Au5d and an Au5p were also observed at 16.7 eV and 67.96 eV, respectively, for this sample. Similarly, in the TrGO/AuNPs spectrum, the Au4f peaks were centered at86.79 eV and 89.83 eV, respectively, while the Au4d peaks were observed at 341.43 eV and 359.71 eV. An Au5d peak was also observed for this sample at 5.77 eV. At% of Au for bare/AuNPs, ErGO/AuNPs, and TrGO/AuNPs were 4.16, 7.38, and 4.08, respectively. This reveals that the ErGO was a better substrate for reducing the AuNPs. Also, the ErGO/AuNPs electrode showed the highest number of AuNPs on the surface, making it the most efficient and promising electrode for further studies. Detailed information about the observed peaks, their positions, the FWHM, and At% is shown in Appendix A in Table A3.

#### 3.2.2. SEM and EDS

**Figure A6:**
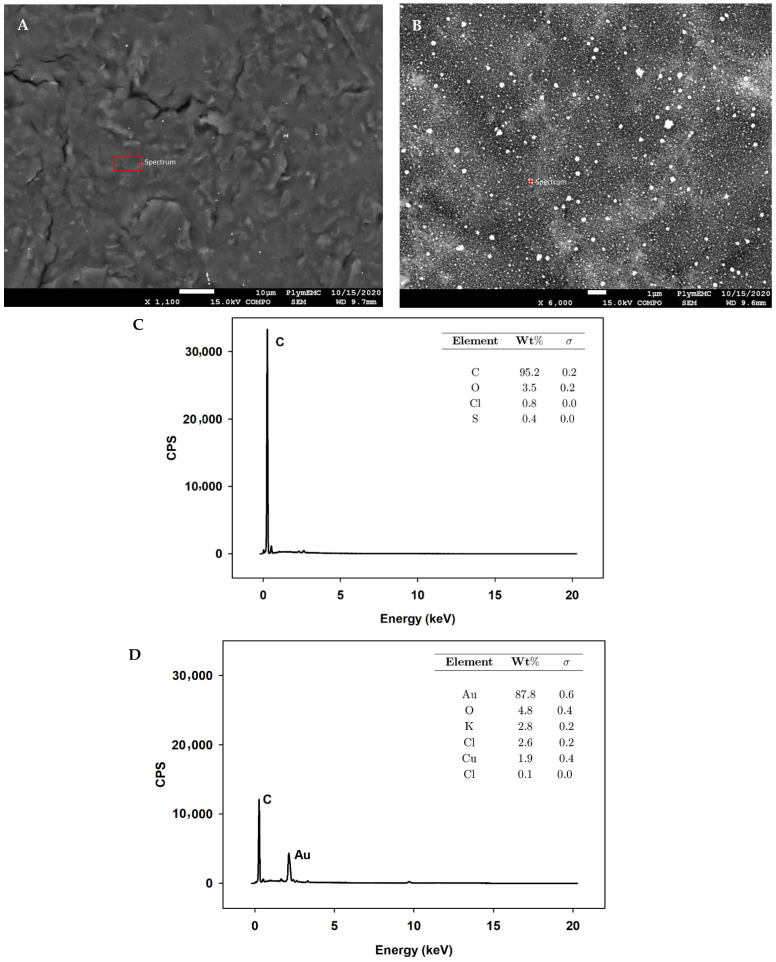
SEM images of ErGO (**A**) and ErGO/AuNPs (**B**) electrodes. EDS spectra of the ErGO (**C**) and ErGO/AuNPs (**D**) electrodes. The inset tables show the present elements, the relative concentration (Wt%) of each element, and their measurement errors (σ).

SEM and EDS were performed to characterize the surface and confirm the presence of gold nanoparticles. Figure A6 shows the SEM images of the ErGO electrode before and after the deposition of the AuNPs on the surface, as well as the EDS spectra of both and the area from which the spectra has been taken. Figure A6A shows the surface of the ErGO electrode exhibiting cracks and wrinkles of 5–20 µm. Figure A6B shows the surface of the ErGO/AuNPs electrode where the AuNPs are homogeneously spread on the surface. The diameter of the nanoparticles is mostly less than 100 nm. EDS spectrographs of the ErGO electrodes before and after the deposition of the AuNPs are shown in Figure A6C,D. The inset tables show the present elements, the relative concentration (Wt%), and the measurement error for each element (σ). EDS measurements confirm the presence of the AuNPs on the surface.

### 3.3. Assay Development

Electrochemical measurements were used to evaluate the biosensor development steps and the performance of the biosensor. CV scans were performed after each preparation step and amperometric detections were conducted after the biosensors were incubated in various concentrations of ds-methylated-DNA (ds-MGMT). The ErGO/AuNPs-modified electrodes were used as the working electrodes for all of the following experiments.

#### 3.3.1. Optimization

**Figure A7:**
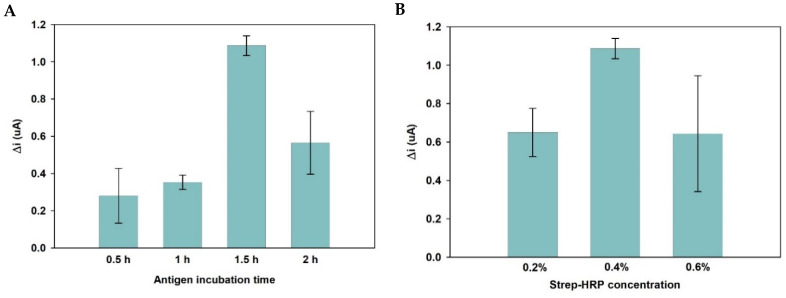
The results of the optimization studies. The incubation time for the optimizing antigen (ds-MGMT) (**A**) and the Streptavidin-HRP concentration (**B**).

The antigen incubation time and the Streptavidin-HRP concentration for the amperometric measurements were optimized prior to incubating the biosensor in various concentrations (Figure A7).

The antigen incubation time was optimized by incubating at least three electrodes in the ds-methylated DNA for either 0.5, 1, 1.5, or 2 h. The results are displayed in Figure A7A where it is shown that the incubation time of 1.5 h exhibits the highest difference in the current before and after adding the H_2_O_2_. Consequently, 1.5 h was chosen as the optimized incubation time for the antigen incubation.

The Streptavidin-HRP concentration was optimized by varying its concentration in PBS. The tested concentrations were 0.2%, 0.4%, and 0.6%, and at least three electrodes were incubated in either of these concentrations for 30 min at room temperature prior to the amperometric measurements. As can be seen in Figure A7B, the 0.4% concentration showed the highest difference in the current before and after adding the H_2_O_2_.Therefore, 0.4% was chosen as the optimized Streptavidin-HRP concentration and was used in further experiments.

#### 3.3.2. Cyclic Voltammetry

**Figure 5:**
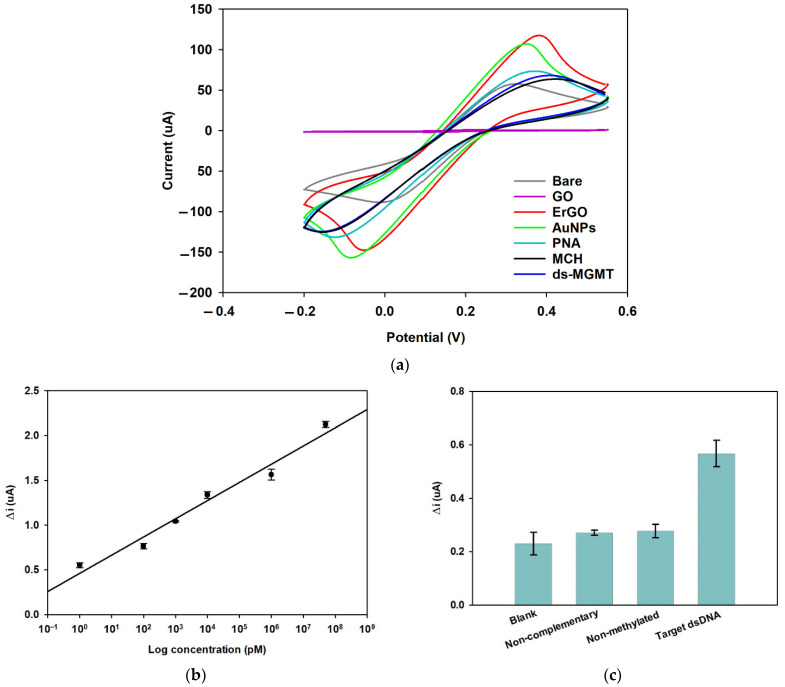
Comparison of CV voltammograms of the various preparation steps of the biosensor: Bare, GO, ErGO, AuNPs, PNA, MCH, and ds-MGMT (**a**). Linear regression studies for the ds-MGMT gene using the amperometric technique. Error bars are the standard deviation of at least three electrodes (**b**). Comparison of the amperometric response of the biosensor in different targets: blank (mouse plasma), the ss-MGMT gene, and the ds-MGMT gene spiked in mouse plasma (**c**).

Figure 5a shows the voltammograms of the various preparation steps of the biosensor (Bare, GO, ErGO, AuNPs, PNA, MCH, and ds-MGMT). As can be seen from the voltammograms, the anodic peak current (i_pa_) of the bare electrode was first seen at 57.5 µA. After drop-coating the surface of the working electrode in GO, the i_pa_ decreased to 1.05 µA due to the non-conductive nature of GO [43]. After the electrochemical reduction of GO, the i_pa_ increased to 117.5 µA. This increase is due to the electrochemical reduction of GO and the production of rGO. RGO is electrically conductive and has a high concentration of charge carriers, mobility, and a high number of available electroactive sites on the surface, facilitating electron transfer [44,45]. After the electrochemical reduction of GO, the i_pa_ decreased to 107.2 µA by reducing the AuNPs on the rGO surface, followed by a further decrease to 73.6 µA after overnight incubation in PNA, confirming the immobilization of the AuNPs and PNA on the surface, respectively. Amine groups (N-terminal of PNA) are able to self-assemble on the AuNPs and form a SAM, decreasing the electron transfer between the electrode and the electrolyte [46,47]. The i_pa_ decreased once more to 63.7 after µA blocking the surface to decrease the chance of non-specific bonding. Finally, the i_pa_ slightly increased to 68.2 µA after the electrode was incubated in target ds-MGMT nucleotides, confirming the presence of the dsDNA on the surface. The dsDNA was captured by the strand invasion of PNA towards the DNA/DNA duplex and the formation of a triple helix [48,49]. Additionally, after the reduction of GO, the peak potential first shifted positively towards higher potentials, followed by a negative shift after the deposition of the AuNPs. Furthermore, a small positive shift in the peak potential was observed after the immobilization of PNA, MCH, and triple formation, which might be due to the spatial blockage and hindered electron transfer on the surface [50].

The cathodic peak currents (i_pc_) of the CV voltammograms showed the same trend as the anodic peak current after each incubation step, with corresponding positive and negative shifts.

#### 3.3.3. Linear Regression

Amperometric detection was used to perform the linear regression studies. As explained in Section 2.4, after the biosensor was incubated in Streptavidin-HRP for 30 min, the amperometric measurements were first performed in the HQ solution for measuring the background signal, followed by the addition of H_2_O_2_ and the measurement of the reduction of the HRP labels. The difference between the background signal and the signal from the HRP reduction (Δi) was plotted as a logarithmic function of the concentration in Figure 5b. As can be seen in this figure, the difference in the current increases with an increase in the concentration due to the presence of more HRP labels; this is correlated with the presence of more ds-MGMT nucleotides. The best fit linear model is y = 0.46ln(x) + 0.20, with R^2^ = 0.96 with the linear range of 1 pM to 50 µM. The LOD was calculated to be 0.86 pM using the equation LOD = 3.3σ/m, where σ is the standard deviation of the amperometric responses of four blank samples and m is the slope of the calibration curve.

#### 3.3.4. Selectivity

The selectivity study was performed with 100 pM of target dsDNA (ds-MGMT) non-complementary as well as non-methylated oligonucleotides spiked in mouse plasma and a blank mouse plasma sample. As can be seen in Figure 5c, there was a significant difference in the responses of the blank and the ds-MGMT samples. Also, the fabricated sensor was able to distinguish between the same concentration of target DNA and the non-complementary and non-methylated DNAs. The higher response of the target DNA means that the biosensor has successfully captured the target DNA, the antibodies have identified the methylated cites on the target DNA, and the reduction of HRP has taken place as described in Section 3.3.3.

#### 3.3.5. Comparison with Other Works

In Table 1, the various parameters of the proposed biosensor, including the working electrode, the bioreceptor, the dynamic range, the LOD, and the measurement techniques, are compared with other electrochemical biosensors so far reported for the detection of DNA methylation. Although most of the works summarized in Table 1 report a better LOD than the results in this work, they all detect single-stranded DNA (ssDNA). In addition, some of the reported biosensors only detect the presence of methylation, insensitive to the gene sequence, while in this work, the presence of the methylation and the sequence of the double-stranded target gene are detected simultaneously.

## 4. Conclusions

A biosensor for the detection of the ds-MGMT gene has been developed in this work using an ErGO/AuNPs-modified electrode. The electrochemical and thermal reductions of GO were also compared. A high C/O ratio was achieved using electrochemical reduction of GO without using any harmful reductants and in a shorter period of time compared to the techniques reported in other papers. The I_D_/I_G_ ratios showed higher numbers of defect sites and a smaller crystallite size of sp^2^ structures in ErGO. After the electro-deposition of the AuNPs, the At% of gold for the ErGO/AuNPs was higher compared to the TrGO/AuNPs. Therefore, the ErGO/AuNPs electrode was used as the base electrode to develop the biosensor. PNA was used to form a SAM layer on the surface via the amine-AuNPs interaction where PNA acts as the bio-recognition element. The linear range was 1 pM to 50 µM and the LOD was calculated to be 0.86 pM without any PCR amplification or bisulfite treatment. Selectivity studies showed that the biosensor is able to distinguish between blank mouse plasma, the target dsDNA, and the non-complementary and non-methylated oligonucleotides spiked in mouse plasma. To the best of our knowledge, this is the first report on using PNA to detect methylated DNA and to capture double-stranded methylated DNA. The sandwich design can be tailor-made to detect other methylated genes, revealing it as basis for clinical applications in diagnostics and a marking it as promising platform for detecting ds-methylated biomarkers.

## Figures and Tables

**Table 1:** A comparison with other biosensing assays to detect methylated DNA.

Electrode	Bioreceptor	Dynamic Range/LOD	Technique	Reference
Gold modified with gold nanoparticles	stem-loop-tetrhedron composite DNA	10^−6^–10 pM 9.326 × 10^−7^ pM	Chronoamperometry	[16]
SPCE and immuno-magnetic beads (MBs)	Anti-5mC	4–2.5 × 10^2^ pM 1 pM	Amperometry	[51]
MoS_2_ Nanosheets	FAM-labeled probe DNA	100–2 × 10^5^ pM 140 pM	Fluorescence	[52]
SPCE modified with rGO and polyvinyl alcohol	Anti-5mC immobilized and DNA probe conjugated with Fe_3_O_4_-citric acid nanocomposites	7 × 10^−4^–140.29 pM 6.31 × 10^−4^ pM	DPV/EIS	[53]
rGO modified with ammuniom hydroxide	Anti5-mC and complementary DNA	0.5–100 pM 0.012 pM	DPV	[36]
AuNPs/ErGO	PNA and anti-5mC	1–5 × 10^7^ pM 0.86 pM	Amperometry	This work

## Data Availability

The data that support the findings of this study are available upon reasonable request from the corresponding author.

## Appendix A

### Appendix A.2. Results and Discussion

#### Appendix A.2.2. XPS

**Table A1:** Detailed information of the XPS peaks, their positions, the FWHM, and the At% for a bare electrode and for the electrodes modified with GO, ErGO, and TrGO. These data are obtained from the XPS survey scans (Figure 2).

Sample	Peak Name	Position	FWHM	At%
Bare	O1s C1s N1s Cl2p	532.10 285.10 400.10 200.10	3.31 3.77 3.10 3.32	19.26 76.49 1.37 2.87
GO	O1s C1s N1s S2p	534.37 287.37 402.37 168.37	3.01 4.50 3.34 3.07	27.98 69.63 1.30 1.10
ErGO	O1s C1s Na1s Cl2p	533.33 285.33 1074.33 199.33	3.89 2.73 2.87 3.48	8.57 90.15 0.44 0.85
TrGO	O1s C1s N1s Cl2p	565.37 312.37 430.37 194.37	3.88 3.06 3.77 3.16	14.65 83.57 1.31 0.47

#### Appendix A.2.3. Raman

**Table A2:** The experimental values of the peak locations, I_D_/I_G_ ratios, and the average crystallite sizes of the sp^2^ lattice (L_a_) of all samples.

Sample	D Peak Location (cm^−1^)	G Peak Location (cm^−1^)	I_D_/I_G_	L_a_ (nm)
Bare	1340	1570	0.77	24.99
GO	1350	1570	0.88	21.84
TrGO	1340	1570	0.89	21.60
ErGO	1350	1570	1.15	16.72

#### Appendix A.2.7. XPS

**Table A3:** Detailed information for the XPS peaks, their position, the FWHM, and the At% for a bare electrode after the deposition of the AuNPs and electrodes modified with GO/AuNPs, ErGO/AuNPs, and TrGO/AuNPs. These data are obtained from XPS survey scans (Figure 4).

Sample	Peak Name	Position	FWHM	At%
Bare/AuNPs	O1s C1s N1s Cl2p S2p Au4f	532.83 284.83 401.83 199.83 168.83 83.83	3.20 3.61 3.27 3.37 3.25 2.50	19.97 72.11 1.56 1.50 0.71 4.16
GO/AuNPs	O1s C1s N1s S2p	531.89 284.89 400.89 168.89	3.00 4.26 2.84 3.01	29.40 63.83 2.76 4.00
ErGO/AuNPs	O1s C1s N1s S2p Au4f	551.00 289.99 418.99 180.99 94.99	3.26 2.77 3.09 2.97 2.80	13.25 74.10 2.89 2.38 7.38
TrGO/AuNPs	O1s C1s N1s S2p Au4f	542.79 298.79 408.79 171.79 86.79	3.43 2.99 3.43 2.96 2.43	17.65 75.06 1.75 1.46 4.08
